# Kinetic Assessment of Mechanical Properties of a Cellulose Board Aged in Mineral Oil and Synthetic Ester

**DOI:** 10.3390/polym13234150

**Published:** 2021-11-27

**Authors:** Cristina Fernández-Diego, Alfredo Ortiz, Isidro A. Carrascal, Inmaculada Fernández, Carlos J. Renedo

**Affiliations:** 1 Electrical and Energy Engineering Department, School of Industrial and Telecomunications Engineering, University of Cantabria, Avenida Los Castros, 39005 Santander, Cantabria, Spain; alfredo.ortiz@unican.es (A.O.); inmaculada.fernandez@unican.es (I.F.); carlos.renedo@unican.es (C.J.R.); 2 LADICIM (Laboratory of Materials Science and Engineering), School of Civil Engineering, University of Cantabria, Avenida Los Castros 44, 39005 Santander, Cantabria, Spain; isidro.carrascal@unican.es

**Keywords:** cellulose board, mineral oil, synthetic ester, kinetic model, power transformer

## Abstract

In oil-immersed power transformers, the insulation system is constituted of a dielectric oil–solid combination. The insulation oil generally used is mineral oil; however, this fluid has started to be substituted by natural and synthetic esters due to their higher biodegradability and flash point. The introduction of a new fluid in the insulation system of power transformers requires kinetic models that can estimate the degradation rate of insulation solids. The aim of this work was to go further in quantifying through different kinetic models the deterioration suffered by a commercial cellulose board (PSP 3055), which is one of the solid materials used in the insulation system of oil-filled transformers. The aging study was extended to cellulose board specimens immersed in two different oils (mineral and synthetic ester). It was obtained that there is a lower degradation when synthetic ester is used in the insulation system. Additionally, it can be concluded that the use of mechanical properties to quantify the degradation of the cellulose board through kinetic models provides information about the different behavior shown by PSP 3055 when different fiber direction angles are considered.

## 1. Introduction

Transformers are critical machines to ensure power systems run properly [1]. In oil-immersed transformers, cellulose solids play a critical role as insulating materials [2], and they are impregnated with insulating oil to resist electric current between conductors. Moreover, the oil is used as a cooling means to evacuate high temperature while transformers are working [3].

During transformers’ operation, the insulation solid degrades slowly to its disintegration [4]. Although the oil also suffers aging, it can be regenerated or replaced easily; therefore, the life of a transformer is determined by its insulation solid.

Due to its high-coolant capability and its excellent dielectric behavior, mineral oil has been used widely as an insulating medium in oil-immersed transformers. Nonetheless, this liquid has impacted the environment because of its non-biodegradability. Moreover, mineral oil possesses poor fire-resistant characteristics, which have encouraged the search for biodegradable choices such as natural and synthetic esters [5,6].

There are works in which the aging characteristics of insulation systems based on esters have been described by focusing on the degradation of fluids [7,8,9] or of solids [10,11,12,13].

Pressboard is a cellulose-based insulation utilized as insulating material for making a variety of components in the design of transformers. This component experiences progressive loss of its mechanical strength, reducing its short-circuit withstand capability [14]. For this reason, the establishment of kinetic models can be an extremely useful tool to estimate the remaining life of insulation solids [15], making it possible to estimate the most suitable moment to replace power transformers [16].

Several works have evaluated the remaining life of insulating solids immersed in different insulation liquids using a wide range of measurements [1,5,15,17,18] in laboratory aging tests. Additionally, the through-thickness compression behavior of mineral oil-impregnated cellulose board [14], the chemical composition and surface topography of impregnated cellulose board samples [19,20], and the change in crystallographic parameters through dilatometry and X-ray diffraction analysis [2] have been studied. All these works in which esters have been considered have concluded that biodegradable dielectric liquids can prolong the life span of cellulose compared with mineral oil.

Although there are studies that have deal with cellulose-board degradation, there are no works in which a detailed kinetic assessment of cellulose-board aged in synthetic ester and mineral oil has been presented. This study aimed at a better understanding of the aging of cellulose-board in synthetic oil-filled transformers. Aging of solid insulation in synthetic ester was also compared with that in mineral oil. Deterioration status of cellulose-board is determined using the degree of polymerization (DP) and mechanical properties such as tensile strength and strain. These mechanical properties can be used as an approximate approach to explain how cellulose board breaks. The application of different kinetic models for characterizing the aging of cellulose-board was extensively studied to determine which one might be the most suitable. Additionally, the kinetic parameters of insulation cellulose-board PSP 3055 aged in mineral and synthetic ester were determined to define the effect of insulation liquid on the degradation rate for the cellulose-board.

## 2. Kinetic Modelling

Synthetic esters are obtained through an esterification reaction using polyols and saturated fatty acid groups [21]. Their different molecular structure in comparison with mineral oil determines their distinct physicochemical properties. One of them has higher moisture solubility in synthetic esters in comparison with mineral oil, which is beneficial because it attracts more moisture from cellulose insulation, increasing the life of the solid insulation. Hydrolysis, which is a decisive chemical reaction involved with water and oil structure, is also critical. The moisture dissolved in the insulation liquid reacts with ester, generating long chain fatty acids, which prevent moisture from moving into the cellulose solid [22].

In this study, different kinetic models (Table 1) were considered to quantify the deterioration suffered by cellulose-board during thermal aging in mineral and synthetic ester. These models were applied using the DP, tensile strength, and strain. The aim was to determine which one is the most suitable to simulate the deterioration of the insulation solid analyzed in this article (PSP 3055).

## 3. Materials and Methods

### 3.1. Material

The insulation systems for this work are composed of insulation oil and a cellulose-board (PSP 3055), whose properties are gathered in Table 2. A high-performance mineral oil and an environment-friendly synthetic ester were used as insulation liquids. Table 3 gives the essential characteristics of both liquids (a naphthenic oil and a synthetic ester).

### 3.2. Thermal Aging

The accelerated tests carried out in the laboratory were fast procedures to simulate the deterioration process quickly, and they can be used to estimate the remaining life under normal operation of the power transformer.

Although there are different properties that impact cellulose aging, in this work we only considered the effect of temperature because as it was demonstrated by Vasovic [29] that temperature has a stronger impact on aging than water content in insulation paper.

To carry out the accelerated thermal aging tests, the cellulose-board was cut into strips of 260 mm × 15 mm. Due to cellulose-board anisotropy, these strips were cut with two different fiber direction angles (machine (MD) and cross direction (CD)).

Secondly, the cellulose-board strips were dried under vacuum at 100 °C for 24 h, then placed them into stainless steel vessels, which were closed and sited in an oven, providing samples with a moisture content of around 2%.

Once the cellulose-board samples were dried, they were placed in oil in stainless steel vessels, which were sealed with nitrogen, and accelerated thermal aging tests were carried out. Aging vessels were placed in an air-circulating oven (UN110, Memmert, Schwabach, Germany) for thermal stressing at different temperatures and duration tests. The aging conditions were 150 °C for 953 h, 130 °C for 2263 h, and 110 °C for 16,155 h of thermal aging. During the aging periods, different samples of cellulose-board were taken out for testing (10 strips for each fiber direction angles). A vessel for each temperature and each oil was prepared by inserting 750 mL of new oil (mineral and synthetic ester) with a nitrogen headspace of 25% by volume.

### 3.3. Characterization

The degree of polymerization (DP) is recognized as an objective detection method to measure the rate of degradation of cellulose products used in transformers’ insulation systems [1,18]. Consequently, in this study, the DP of each group of specimens of PSP 3055 was measured following the viscometric method of the standard ASTM D4243.

The tensile test is also recognized as an objective detection method to evaluate the mechanical strength of cellulose-board when a specimen is clamped in an axial loading frame. The data obtained from this test (load and displacement) were used to determine tensile strength (σ_R_) and strain under ultimate strength (ε_cm_) because they are two parameters that provide suitable information about the real deterioration suffered by the insulation solid [12].

In this work, a universal servo hydraulic test machine (Model ME-405-1, SERVOSIS, Madrid, Spain) was used to carry out the tensile tests according to ISO 1924-2.

## 4. Results and Discussion

This section shows the degradation suffered by cellulose-board aged in mineral and synthetic ester.

### 4.1. DP and Mechanical Properties during Thermal Aging

**Figure 1:**
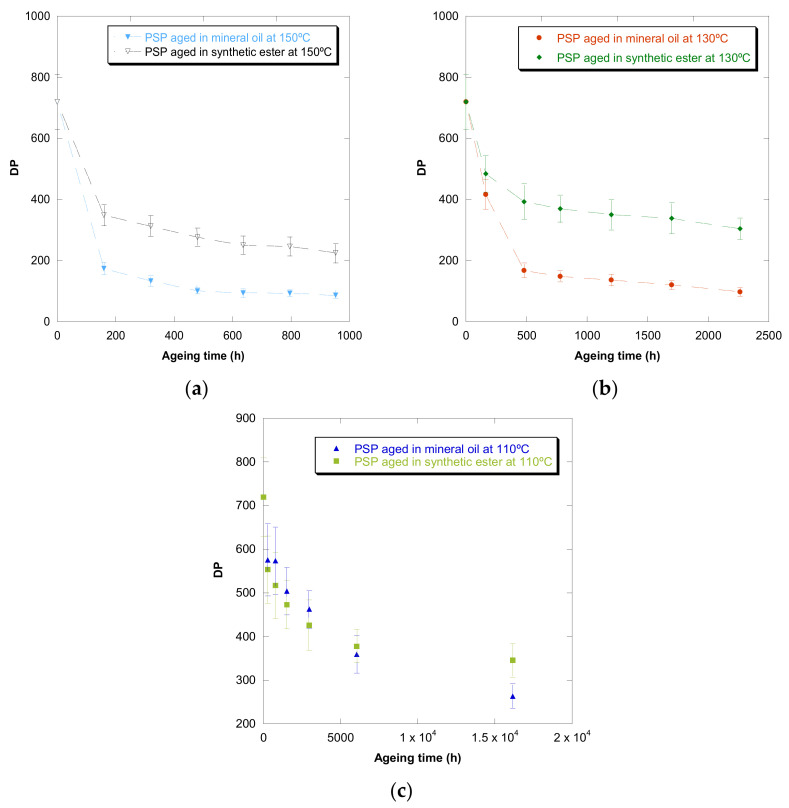
Evolution of the DP as a function of temperature for cellulose-board PSP 3055 aged in mineral oil and in synthetic ester at 150 °C (**a**), 130 °C (**b**), and 110 °C (**c**).

**Figure 2:**
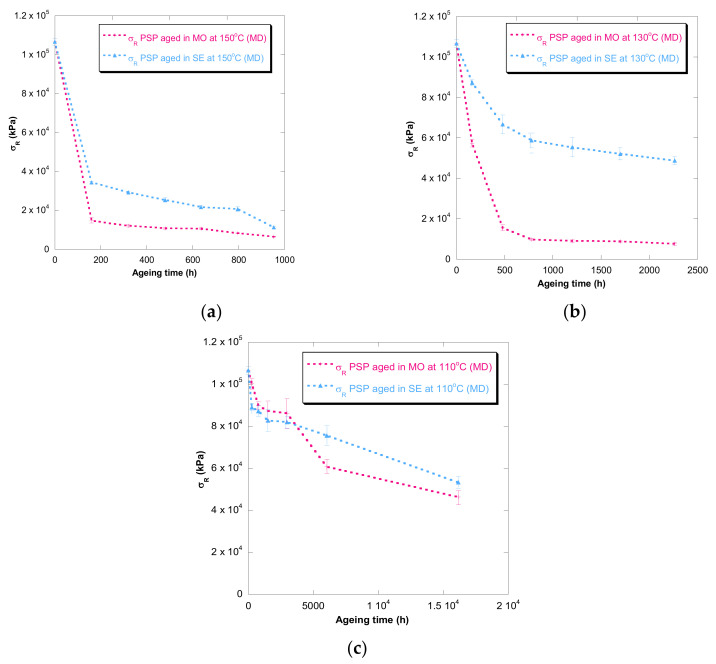
Evolution of the σ_R_ in MD as a function of temperature for cellulose-board PSP 3055 aged in mineral oil and in synthetic ester at 150 °C (**a**), 130 °C (**b**), and 110 °C (**c**).

**Figure 3:**
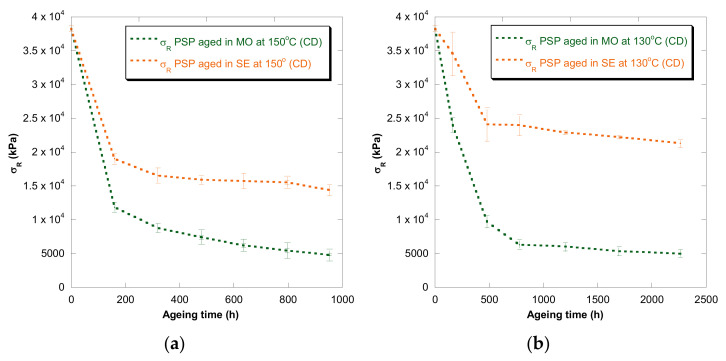

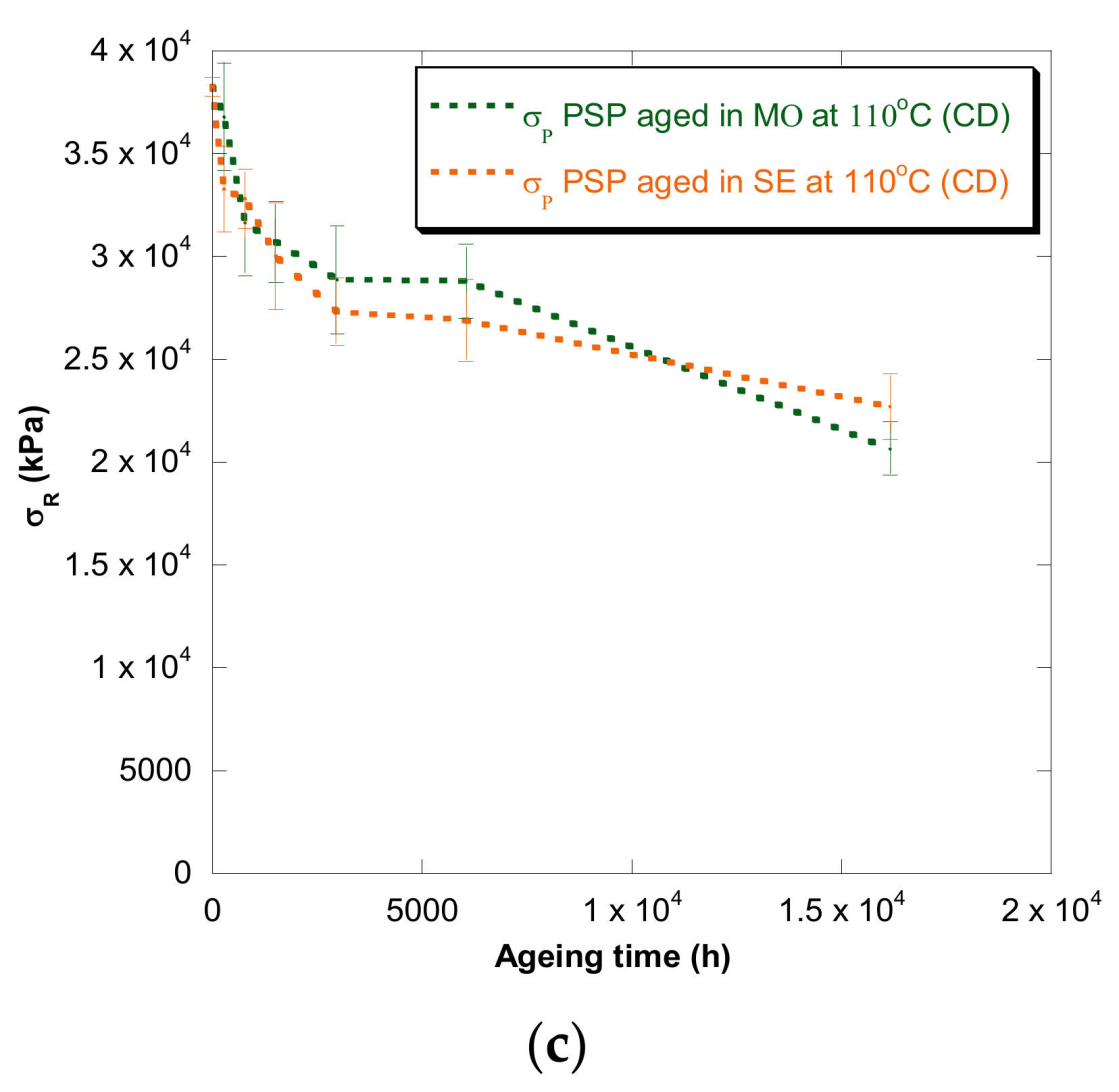
Evolution of the σ_R_ in CD as a function of temperature for cellulose-board PSP 3055 aged in mineral oil and in synthetic ester at 150 °C (**a**), 130 °C (**b**), and 110 °C (**c**).

**Figure 4:**
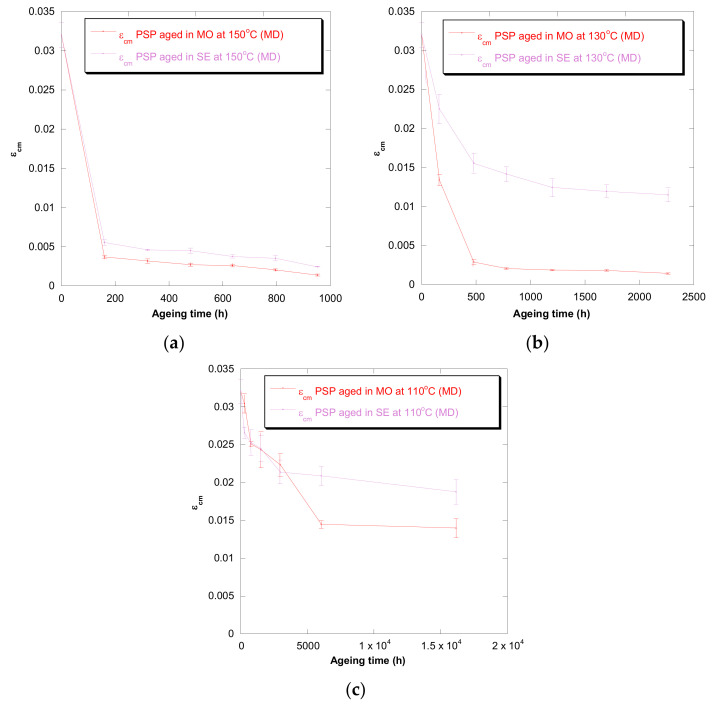
Evolution of the ε_cm_ in MD as a function of temperature for cellulose-board PSP 3055 aged in mineral oil and in synthetic ester at 150 °C (**a**), 130 °C (**b**), and 110 °C (**c**).

**Figure 5:**
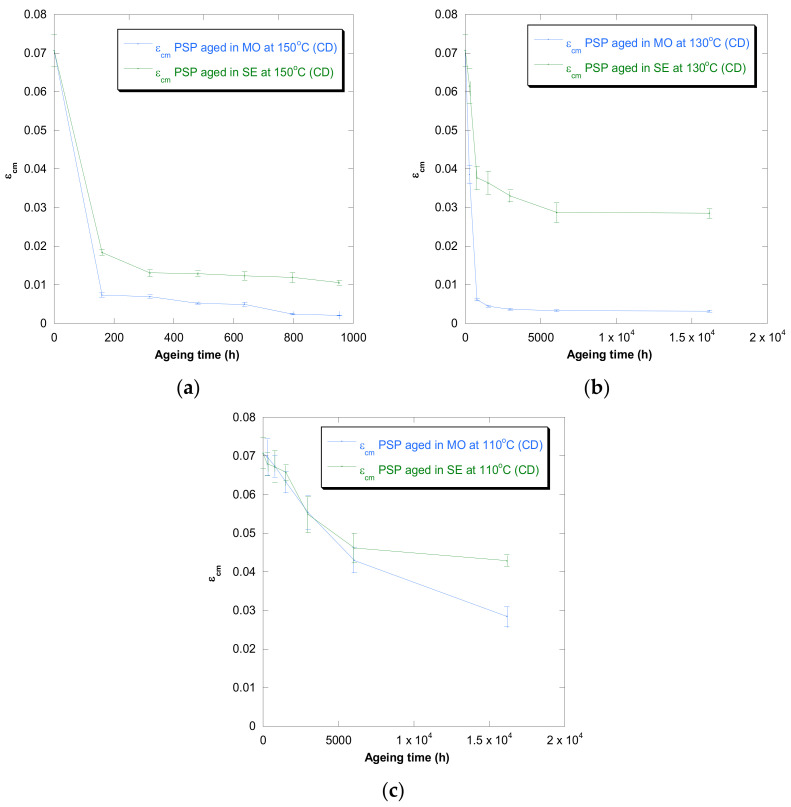
Evolution of the ε_cm_ in CD as a function of temperature for cellulose-board PSP 3055 aged in mineral oil and in synthetic ester at 150 °C (**a**), 130 °C (**b**), and 110 °C (**c**).

Figure 1, Figure 2, Figure 3, Figure 4 and Figure 5 show DP, tensile strength (σ_R_), and strain (ε_cm_) curves for cellulose-board artificially aged at three different temperatures. Bulleted lists look like this:

The results showed that the damage increased over time and this increase became faster with the rise of temperature in both insulation liquids (mineral oil and synthetic ester). At the three temperatures, it was observed that the synthetic ester preserved better the state of the cellulose-board for the same aging time. The deterioration suffered by PSP, as was explained previously, is suggested to be distinct due to the different moisture equilibriums between oil and paper and hydrolysis reactions. However, this protective effect of synthetic ester seemed to be less significant as the temperature was reduced. The reason for that is unclear, but it might be associated with the water generated as a by-product of the deterioration, which played a more critical role in the degradation of the cellulose at higher temperatures, raising the rate of bond scission [30]. Since, in insulation systems based on synthetic ester/solid, the moisture tends to remain in the oil, obtaining an insulation solid drier, the effect of synthetic ester is more beneficial when the temperature is higher.

In the case of the mechanical properties considered in this work (σ_R_ and ε_cm_), the observed behavior was similar to that found for DP. However, when these kinds of properties are evaluated, their values depend on the orientation of the paper’s fibers with respect to the applied load during the tensile test due to cellulose-board’s anisotropy. The values of σ_R_, when the PSP fibers are in the same direction as that with which the test machine applies the load (MD), have been found to be two times the ones measured when the fibers are in a cross direction (CD) to the test machine, while the ε_cm_ is half approximately. This different behavior must be taken into consideration since, depending on the way the material is placed and the voltages are applied during the transformer operation, the cellulose-board resistance can be considerably affected. When a load is applied with fibers aligned, the fibers length is higher, and this contributes to an increase in the mechanical strength of the insulation solid.

For all the properties (DP, σ_R,_ and ε_cm_), it was found that there was an important linear decrease at the beginning of thermal aging and a later stabilization of the values. Additionally, the loss of mechanical properties was observed in a similar way when fiber orientation was MD and CD.

Once the experimental values of the DP, σ_R_, ε_cm,_ and the uncertainties were obtained, the correlation between these experimental data and the adjustment performed according to different kinetic models was analyzed. Additionally, the propagation of uncertainties in kinetic parameters and the coefficient of determination are summarized in the following section.

### 4.2. Ekenstam Kinetic Model

**Figure 6:**
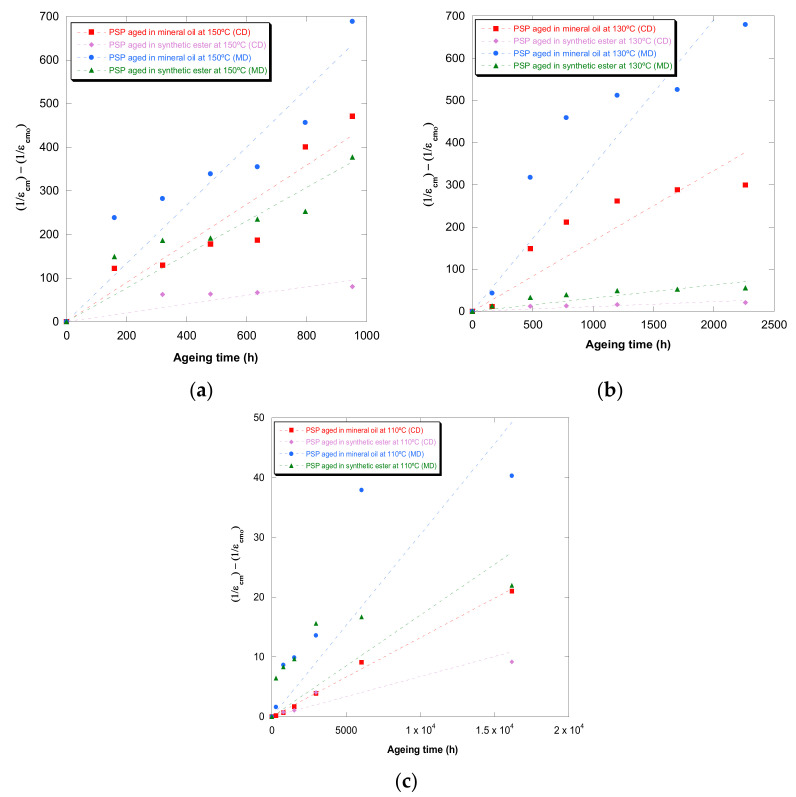
Evolution of the (1/ε_cm_ − 1/ε_cm0_) in MD and CD as a function of temperature for pressboard PSP 3055 aged in mineral oil and in synthetic ester at 150 °C (**a**), 130 °C (**b**), and 110 °C (**c**) and Ekenstam’s function.

Ekenstam defined a kinetic model based on first-order chemical kinetic considerations (Table 1). The graph of (1/ε_cm_ − 1/ε_cm0_) against time using experimental data and the model proposed by Ekenstam are plotted in Figure 6 for PSP 3055 at three different temperatures.

Similar representations were carried out for (1/DP − 1/DP_0_) and (1/σ_R_ − 1/σ_R0_). These graphs were used to determine the constant rate of reaction for each temperature and insulation liquid, and their values are gathered in Table 4.

Although the values of the coefficients of determination (R2), which were obtained, were high enough for some aging conditions in mineral oil, they reveal there was not a suitable agreement between experimental values (DP, σ_R,_ and ε_cm_) and the Ekenstam model when the cellulose-board was aged in synthetic ester.

It was verified that the constant rates of reaction increased considerably with the aging temperature. When the constant rates of mineral oil were compared with those calculated for synthetic ester, it was verified that these constants were higher. Therefore, the insulation liquid impacted the solid degradation substantially.

### 4.3. Emsley Kinetic Model

The equation proposed by Ekenstam was modified by Emsley et al. [24] because they observed that, whether the DP value of insulation paper decreases to a certain value, its behavior gradually deviates from the first-order kinetic (Table 1).

The change in rate constant seemed to be related with the inhomogeneous composition of the cellulosic solid (cellulose, lignin, and hemicelluloses). Additionally, it is known that the amorphous regions of cellulose are more reactive than the crystalline regions in which the degradation is very slow due to the higher order in cellulose chains, which reduces the accessibility of aging agents [31].

**Figure 7:**
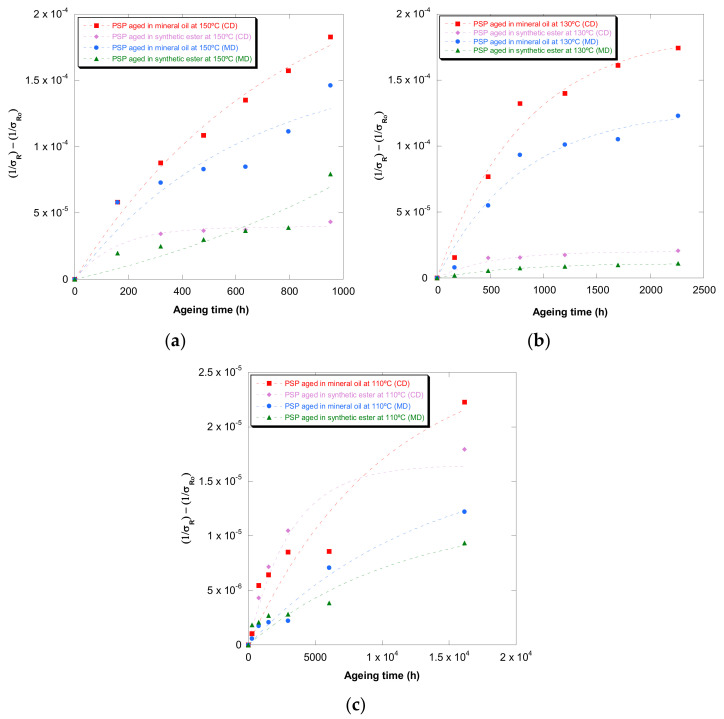
Evolution of the (1/ε_cm_ − 1/ε_cm0_) in MD and CD as a function of temperature for pressboard PSP 3055 aged in mineral oil and in synthetic ester at 150 °C (**a**), 130 °C (**b**), and 110 °C (**c**) and Emsley’s function.

The constants of the Emsley et al. model [24] were obtained through the representation of (1/σ_R_ − 1/σ_R0_), (1/ε_cm_ − 1/ε_cm0_), and (1/DP − 1/DP_0_) against time for PSP 3055 at the aging temperatures and the studied insulation liquids. In Figure 7 is shown the shape of the curves of Emsley’s model based on tensile strength. Similar representations were obtained for ε_cm_ and DP.

The values of both constants (k_10_ and k_2_) are gathered in Table 5. This kinetic model had a high agreement with the experimental measures, with all the coefficients of determination (R2) being higher than 0.84. The three properties considered in this study seemed to be suitable to estimate PSP 3055 deterioration using this kinetic model.

As previously, it was verified that the constants of this kinetic model were highly influenced by the aging temperature and type of oil.

### 4.4. Zervos’ Kinetic Model

Although this model was defined from the study of pure cotton cellulose, it was used to model the deterioration of cellulose-board measured in this work.

**Figure 8:**
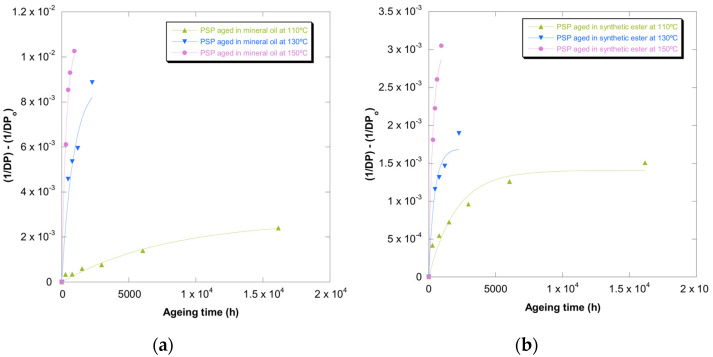
Evolution of the (1/DP -1/DP0) as a function of temperature for pressboard PSP 3055 aged at 150 °C, 130 °C, and 110 °C, and Zervos’ function, in mineral oil (**a**) and in synthetic ester (**b**).

The constants of the Zervos model [25] were obtained through the representation of (1/σ_R_ – 1/σ_R0_), (1/ε_cm_ – 1/ε_cm0_), and (1/DP – 1/DP_0_) against time, as with previous models. The curves obtained from the measures of the DP of the pressboard are shown in Figure 8. This representation was also done for ε_cm_ and σ_R_.

The values of the calculated constants (k and a) are shown in Table 6. This kinetic model had a high agreement with the experimental measures, with the coefficients of determination (R2) being slightly lower than the ones obtained with the Emsley model.

Both mechanical properties (ε_cm_ and σ_R_) and the DP allowed us to estimate the degradation of the PSP 3055 through this mathematical approach.

### 4.5. Calvini Kinetic Model

The kinetic models used in previous sections only dealt with the extent of cellulose degradation in terms of the ratio of broken glucose units to the total glucose units of a cellulose chain (SFCU = 1/DP − 1/DP_0_). As was pointed out by Calvini and Gorassini [26], every experimental uncertainty about the actual values of DP_0_ and DP is amplified in the determination of the cellulosic solids’ degradation. For this reason, those authors introduced the concept of S = (DP_0_/DP) − 1, which represents the average number of chain scissions per cellulose chain unit during the time of degradation. Additionally, they considered the so-called levelling-off value (LODP), which means the beginning of a third, very slow degradation stage of the cellulose. This stage can be found only with very drastic degradations, in a range of DP below the so-called levelling-off value, where nothing but crystallites remain and the tensile strength of cellulose is lost forever. Before this third stage, there are two distinct stages: a fast initial attack to the so-called weak links followed by a slower one to the amorphous fraction.

To apply this model, in this work we established for LODP a DP of 200, ε_cm,_ and σ_R_ (25% of initial value) [32].

**Figure 9:**
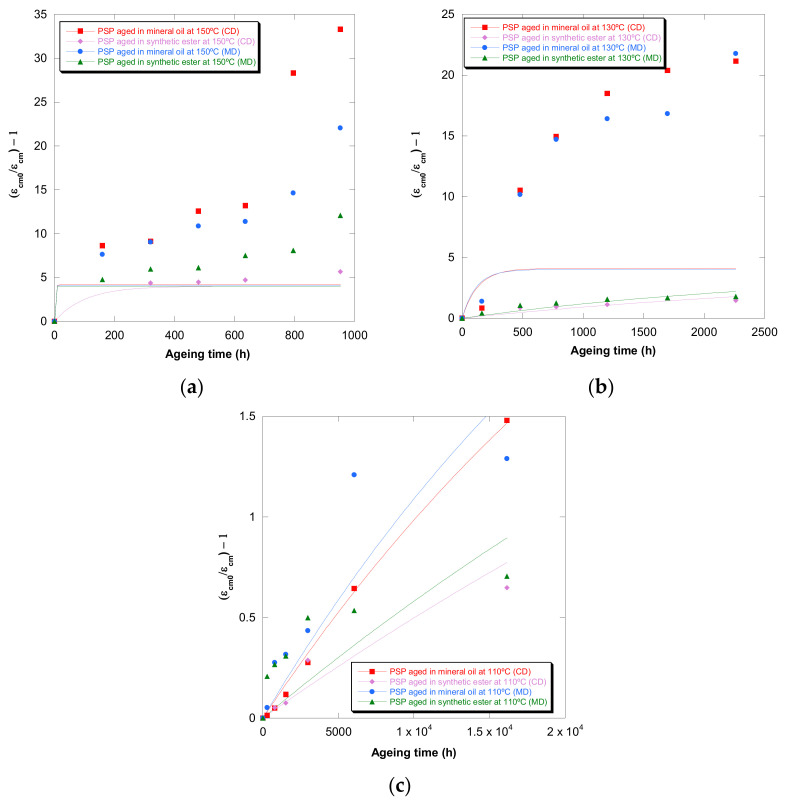
Evolution of the (ε_cm0_/ε_cm_) − 1 in MD and CD as a function of temperature for pressboard PSP 3055 aged in mineral oil and in synthetic ester at 150 °C (**a**), 130 °C (**b**), and 110 °C (**c**) and Calvini’s function.

The constant of the Calvini and Gorassini model [26] was obtained through the representation of (σ_R0_/σ_R_ − 1), (ε_cm0_/ε_cm_ − 1), and (DP_0_/DP − 1) against time. The curves based on strain are gathered in Figure 9. The same representation was carried out for σ_R_ and DP.

The values of the constant rate of reaction (k) are gathered in Table 7. It can be observed that there was not a suitable agreement between experimental values (DP, σ_R,_ and ε_cm_) and the Calvini mathematical approach.

On the other hand, it was found that the values of DP, σ_R,_ and ε_cm_ selected as levelling off possessed a huge impact on the R2. Moreover, this impact depended on the aging temperature, insulation liquid, and property used to quantify the deterioration suffered by PSP 3055. Therefore, this mathematical approach seemed to be influenced by different variables, which makes its application more complicated in comparison with other models.

### 4.6. Weidmann Kinetic Model

This was one of the earliest models formulated empirically by researchers at Weidmann [27].

**Figure 10:**
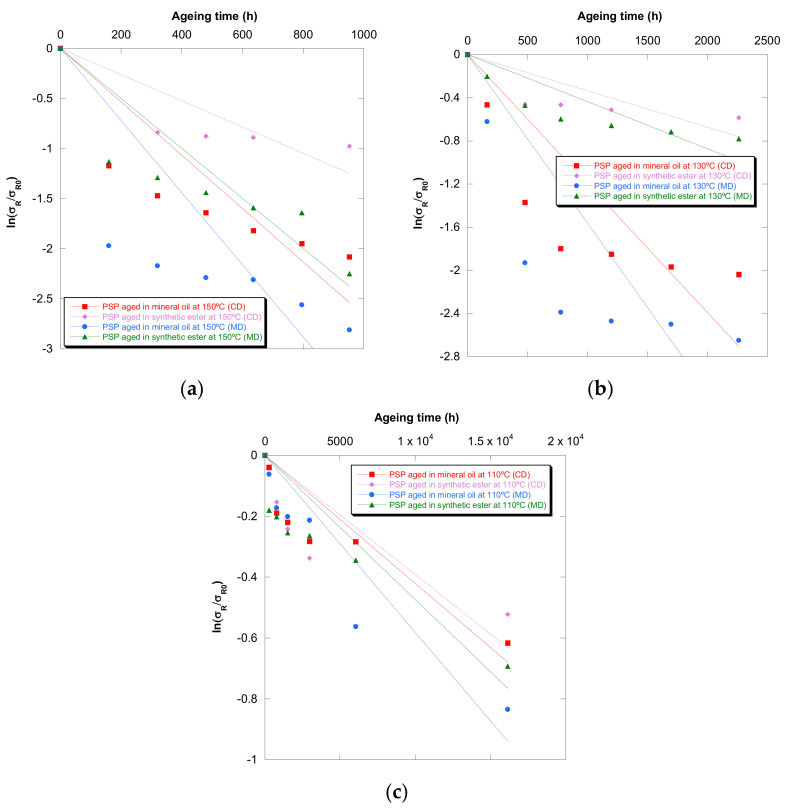
Evolution of the ln (σ_R_/σ_R0_) in MD and CD as a function of temperature for pressboard PSP 3055 aged in mineral oil and in synthetic ester at 150 °C (**a**), 130 °C (**b**), and 110 °C (**c**) and Weidmann’s function.

The representation of ln(σ_R_/σ_R0_), ln (ε_cm_/ε_cm0_), and ln (DP/DP_0_) against time allowed us to obtain the values of the coefficient of aging of this model [27]. Figure 10 displays the curves based on tensile strength. The curves based on strain and DP were also done.

The values of the coefficient of aging are shown in Table 8. The coefficients of determination (R2) were low; in consequence, the Weidmann model did not allow us to make a good estimation of the cellulose-board deterioration.

### 4.7. IEEE Kinetic Model

**Figure 11:**
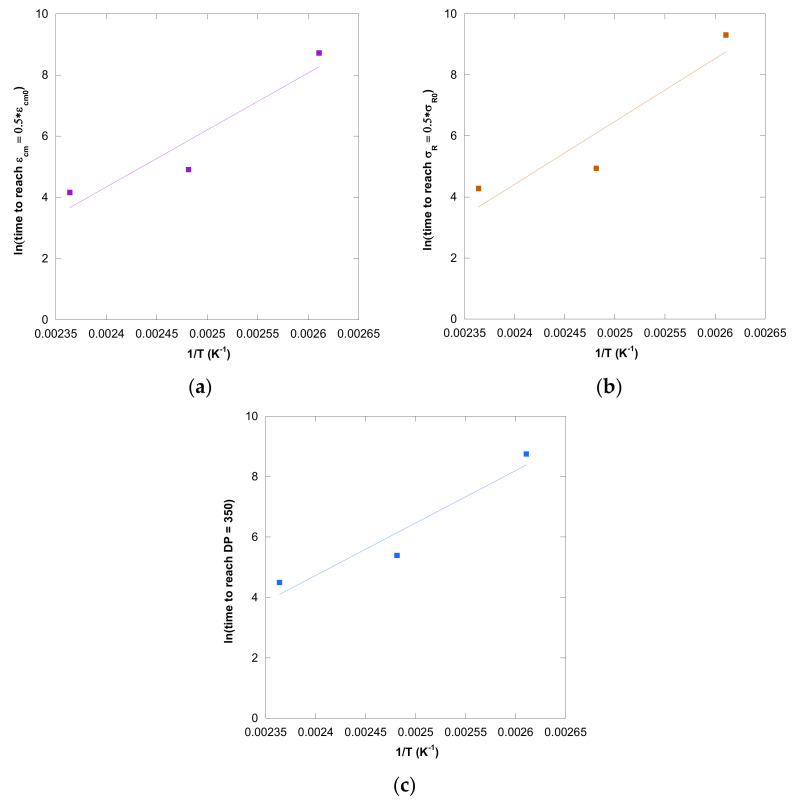
Evolution of the ln (time) vs. inverse temperature in CD for pressboard PSP 3055 aged in mineral oil considering: ε_cm_ (**a**), σ_R_ (**b**), and DP (**c**).

The constants of this mathematical approach were calculated using the representation of ln (time required to reach the end-of-life criteria) versus inverse temperature (1/T), resulting in a linear curve, as can be seen in the Figure 11. To guarantee that the end-of-life criteria was reached for all the conditions studied in this article, the following aging stages were established as points of comparison: DP = 350, σ_R_ = 0.50 ∗ σ_R0,_ and ε_cm_ = 0.50 ∗ ε_cm0_.

The values of the constants of the IEEE mathematical approach are displayed in Table 9. Although the calculated coefficients of determination (R2) were high, they reduced their values when the insulation solid underwent a greater deterioration, which happened when the samples of PSP 3055 were aged in mineral oil. On the other hand, it was necessary to highlight the effect that the criterion established as end of life had on the results. In this work, the values of DP, σ_R,_ and ε_cm_ established as points of comparison were quite high, being located approximately in the fast-initial attack to the insulation solid during thermal aging. Therefore, it would be convenient to carry out longer aging tests at low temperatures to apply end-of-life criteria in which the material would have experienced higher deterioration, obtaining more information about the suitability of this model.

## 5. Conclusions

Different kinetic models based on the variation of degree of polymerization (DP), tensile strength (σ_R_), and strain (ε_cm_) were used to quantify the deterioration of cellulose-board PSP 3055 aged in mineral oil and synthetic ester.

The measurement of DP and the mechanical properties (σ_R_ and ε_cm_) showed the effect of the insulation liquid on the degradation suffered by cellulose-board during thermal aging, concluding there was a lower degradation when synthetic ester was used in the insulation system.

It was been obtained that the kinetic models proposed by Emsley [24] and Zervos [25] allowed us to obtain the highest correlation coefficients in both mineral and synthetic ester. Moreover, it was found that the suitability and accuracy of some of the models applied in this work [26,28] depended on the levelling-off value established for the constant rate calculation or the end-of-life criteria established as a point of comparison, which made their implementation more challenging and complex.

In all the kinetic models considered in this paper, their characteristic parameters, as well as their correlation coefficients, suffered variations in their values that were generally lower than 10% when the uncertainties in measurements were considered, which validated the robustness of the observed trends during thermal aging in both insulation liquids.

Additionally, it can be concluded that the use of mechanical properties to quantify the degradation of the cellulose-board evaluated in this study through a kinetic model provided information about the different behavior shown by PSP 3055 when different fiber direction angles (MD and CD) were considered. Therefore, kinetic models based on mechanical properties make possible the assessment of the effect of cellulose-board anisotropy, which cannot be considered when DP is used to evaluate its aging.

## Figures and Tables

**Table 1:** Kinetic models of cellulose solid deterioration.

**Ekenstam Kinetic model**		
$(1/DP)-(1/DP_{0})=k*t$	(1)	[23]
where $DP_{0}$ is the Number of average degree of polymerization at the time of 0, DP is the Number of average degree of polymerization at the time of t, k is the Constant rate of reaction, and t is the Aging time (h).		
**Emsley kinetic model**		
$(1/DP)-(1/DP_{0})=(k_{1_{0}}/k_{2})*\left(1-exp\left(-k_{2}*t\right)\right)$	(2)	[24]
where $K_{1_{0}}$ and $K_{2}$ are Temperature-dependent constants.		
**Zervos Kinetic model**		
$(1/DP)-(1/DP_{0})=(a/100)*\left(2^{k*t}-1\right)$	(3)	[25]
where k and a are Temperature-dependent constants.		
**Calvini Kinetic model**		
$(DP_{0}/DP)-1=(DP_{0}/LODP)*\left(1-exp\left(-k*t\right)\right)$	(4)	[26]
where LODP is the Leveling-off degree of polymerization.		
**Weidmann Kinetic model**		
$TS=TS_{0}*\left(exp\left(-C_{TS}\left(T\right)*t\right)\right)$	(5)	[27]
$DP=DP_{0}*\left(exp\left(-C_{DP}\left(T\right)*t\right)\right)$	(6)	
where TS is the Tensile strength aged, $TS_{0}$ is the Tensile strength unaged and $C_{TS}$ and $C_{DP}$ are Coefficients of aging.		
**IEEE Kinetic model**		
$\ln L\left(T\right)=A+\left(B/T\right)\;or\;L\left(T\right)=A*exp\left(B/T\right)$	(7)	[28]
where ln $L\left(T\right)$ is the Lifetime of the power transformer and A and B are Temperature-dependent constants.		

**Table 2:** PSP 3055 properties.

Property	Value
Apparent density (kg/m^3^)	1100 ± 6.20%
Degree of polymerization (before impregnation)	750 ± 9.50%
pH of aqueous extract	7.20 ± 8.41%
Moisture content (%)	<8 ± 4.32%
Ash content (%)	0.30 ± 8.22%
Conductivity of aqueous extract (mS/m)	2.0 ± 9.70%
Electric strength in air unfolded (kV/mm)	7 ± 3.11%
Electric strength in oil (kV/mm)	50 ± 5.40%

**Table 3:** Properties of commercial oils analyzed.

Property	Standard	Mineral Oil (MO)	Synthetic Ester (SE)
Viscosity, 40 °C (mm^2^/s)	ISO 3104, ASTM D445	10.30 ± 2.90%	29 ± 12.70%
Density, 20 °C (kg/dm^3^)	ISO 3675, ASTM D4052	0.84 ± 6.50%	0.97 ± 3.20%
Pour point (°C)	ISO 3016, ASTM D97	−63 ± 9.70%	−56 ± 10.00%
Flash point (°C)	ISO 2719, ASTM D92	160 ± 8.60%	260 ± 9.20%
Water content (mg/kg)	IEC 60814	15 ± 8.70%	50 ± 11.50%
Breakdown voltage (kV)	IEC 60156	>70 ± 7.30%	>75 ± 12.40%
Acidity (mg KOH/g)	IEC 62021.1, ASTM D974	<0.01 ± 1.70%	<0.03 ± 3.20%
Dielectric dissipation factor (90 °C)	IEC 60247	7 × 10^−4^ ± 8.20%	<8 × 10^−3^ ± 12.80%

**Table 4:** Constant rates of reaction for Ekenstam model based on DP, ε_cm,_ and σ_R_ for different insulation oils.

$(1/Property\;\left(DP,\;\varepsilon_{cm},\;\sigma_{R}\right)-(1/Property{\;}_{0}\;\left(DP,\;\varepsilon_{cm},\;\sigma_{R}\right))=k\times t$				
	Mineral oil		Synthetic ester	
Temperature (°C)	k (DP)	R2	k (DP)	R2
150	1.30 × 10^−5^ ± 10.47%	0.74 ± 0.82%	3.72 × 10^−6^ ± 11.29%	0.72 ± 9.33%
130	4.41 × 10^−6^ ± 11.96%	0.80 ± 0.06%	1.01 × 10^−6^ ± 12.07%	0.49 ± 4.84%
110	1.65 × 10^−7^ ± 8.76%	0.86 ± 0.55%	1.17 × 10^−7^ ± 8.26%	0.07 ± 12.38%
Temperature (°C)	k (ε_cm_) _CD_	R2	k (ε_cm_) _CD_	R2
150	4.48 × 10^−1^ ± 6.31%	0.89 ± 0.47%	1.01 × 10^−1^ ± 7.29%	0.53 ± 8.03%
130	1.67 × 10^−1^ ± 7.02%	0.77 ± 2.28%	1.15 × 10^−2^ ± 6.53%	0.76 ± 4.86%
110	1.32 × 10^−3^ ± 7.62%	0.99 ± 4.22%	6.73 × 10^−4^ ± 3.89%	0.77 ± 9.04%
Temperature (°C)	k (ε_cm_) _MD_	R2	k (ε_cm_) _MD_	R2
150	6.67 × 10^−1^ ± 6.83%	0.87 ± 0.34%	3.84 × 10^−1^ ± 4.69%	0.81 ± 2.70%
130	3.46 × 10^−1^ ± 5.87%	0.79 ± 1.01%	3.13 × 10^−2^ ± 8.99%	0.63 ± 3.56%
110	3.05 × 10^−3^ ± 9.08%	0.66 ± 11.07%	1.70 × 10^−3^ ± 11.76%	0.03 ± 7.65%
Temperature (°C)	k (σ_R_) _CD_	R2	k (σ_R_) _CD_	R2
150	2.06 × 10^−7^ ± 5.13%	0.94 ± 1.25%	5.55 × 10^−8^ ± 6.50%	0.37 ± 1.94%
130	9.49 × 10^−8^ ± 11.82%	0.77 ± 0.06%	1.16 × 10^−8^ ± 5.54%	0.54 ± 5.43%
110	1.46 × 10^−9^ ± 8.74%	0.83 ± 6.12%	1.30 × 10^−9^ ± 9.16%	0.45 ± 6.10%
Temperature (°C)	k (σ_R_) _MD_	R2	k (σ_R_) _MD_	R2
150	1.54 × 10^−7^ ± 3.89%	0.83 ± 3.32%	6.79 × 10^−8^ ± 4.62%	0.83 ± 0.82%
130	6.58 × 10^−8^ ± 7.86%	0.75 ± 2.58%	5.94 × 10^−9^ ± 9.22%	0.74 ± 7.06%
110	8.13 × 10^−10^ ± 11.28%	0.93 ± 0.48%	6.12 × 10^−6^ ± 9.98%	0.81 ± 1.43%

**Table 5:** Constant rates of reaction for the Emsley model based on DP, ε_cm,_ and σ_R_ for different insulation oils.

$(1/Property\;\left(DP,\;\varepsilon_{cm},\;\sigma_{R}\right)-(1/Property{\;}_{0}\;\left(DP,\;\varepsilon_{cm},\;\sigma_{R}\right))=(k_{1_{0}}/k_{2})\times \left(1-exp\left(-k_{2}\times t\right)\right)$						
	Mineral oil				Synthetic ester	
Temperature (°C)	k_10_ (DP)	k_2_ (DP)	R2	k_10_ (DP)	k_2_ (DP)	R2
150	3.23 × 10^−5^ ± 9.31%	3.02 × 10^−3^ ± 1.23%	0.99 ± 0.40%	9.59 × 10^−6^ ± 0.78%	3.18 × 10^−3^ ± 8.30%	0.98 ± 0.19%
130	9.97 × 10^−6^ ± 10.35%	1.12 × 10^−3^ ± 0.97%	0.96 ± 0.52%	3.85 × 10^−6^ ± 9.80%	2.28 × 10^−3^ ± 3.46%	0.96 ± 1.82%
110	3.43 × 10^−7^ ± 8.26%	1.25 × 10^−4^ ± 0.94%	0.97 ± 0.51%	5.03 × 10^−7^ ± 10.20%	3.36 × 10^−4^ ± 9.96%	0.91 ± 4.66%
Temperature (°C)	k_10_ (ε_cm_) _CD_	k_2_ (ε_cm_) _CD_	R2	k_10_ (ε_cm_) _CD_	k_2_ (ε_cm_) _CD_	R2
150	2.89 × 10^−1^ ± 10.54%	−1.05 × 10^−3^ ± 9.69%	0.92 ± 0.13%	3.59 × 10^−1^ ± 0.66%	4.84 × 10^−3^ ± 8.61%	0.98 ± 1.16%
130	3.95 × 10^−1^ ± 4.22%	1.20 × 10^−3^ ± 4.46%	0.97 ± 0.24%	2.79 × 10^−2^ ± 10.93%	1.25 × 10^−3^ ± 8.46%	0.97 ± 0.61%
110	1.49 × 10^−3^ ± 6.23%	1.66 × 10^−5^ ± 9.54%	0.99 ± 1.30%	1.74 × 10^−3^ ± 10.85%	1.72 × 10^−4^ ± 10.16%	0.96 ± 1.91%
Temperature (°C)	k_10_ (ε_cm_) _MD_	k_2_ (ε_cm_) _MD_	R2	k_10_ (ε_cm_) _MD_	k_2_ (ε_cm_) _MD_	R2
150	8.27 × 10^−1^ ± 5.97%	5.90 × 10^−4^ ± 4.49%	0.87 ± 0.42%	6.60 × 10^−1^ ± 8.20%	1.61 × 10^−3^ ± 7.86%	0.87 ± 3.89%
130	8.03 × 10^−1^ ± 7.84%	1.17 × 10^−3^ ± 3.25%	0.96 ± 0.04%	9.51 × 10^−2^ ± 9.56%	1.69 × 10^−3^ ± 3.64%	0.99 ± 0.08%
110	8.94 × 10^−3^ ± 3.42%	2.04 × 10^−4^ ± 7.71%	0.94 ± 4.03%	1.07 × 10^−2^ ± 7.80%	5.43 × 10^−4^ ± 9.44%	0.91 ± 2.72%
Temperature (°C)	k_10_ (σ_R_) _CD_	k_2_ (σ_R_) _CD_	R2	k_10_ (σ_R_) _CD_	k_2_ (σ_R_) _CD_	R2
150	3.24 × 10^−7^ ± 10.19%	1.31 × 10^−3^ ± 8.32%	0.98 ± 0.11%	2.47 × 10^−7^ ± 5.73%	6.22 × 10^−3^ ± 0.99%	0.99 ± 0.62%
130	2.26 × 10^−7^ ± 9.60%	1.21 × 10^−3^ ± 0.39%	0.98 ± 0.23%	4.07 × 10^−8^ ± 9.31%	2.02 × 10^−3^ ± 4.43%	0.95 ± 0.73%
110	2.69 × 10^−9^ ± 8.53%	9.98 × 10^−5^ ± 9.37%	0.89 ± 7.73%	5.17 × 10^−9^ ± 8.32%	3.13 × 10^−4^ ± 7.24%	0.91 ± 2.08%
Temperature (°C)	k_10_ (σ_R_) _MD_	k_2_ (σ_R_) _MD_	R2	k_10_ (σ_R_) _MD_	k_2_ (σ_R_) _MD_	R2
150	2.66 × 10^−7^ ± 10.20%	1.63 × 10^−3^ ± 5.20%	0.90 ± 0.67%	4.76 × 10^−8^ ± 8.84%	−8.56 × 10^−4^ ± 10.11%	0.85 ± 0.34%
130	1.92 × 10^−7^ ± 8.20%	1.26 × 10^−3^ ± 3.49%	0.97 ± 0.05%	1.54 × 10^−8^ ± 8.13%	1.37 × 10^−3^ ± 8.82%	0.99 ± 0.63%
110	1.32 × 10^−9^ ± 8.16%	7.63 × 10^−5^ ± 8.41%	0.97 ± 1.59%	1.07 × 10^−9^ ± 9.17%	8.90 × 10^−5^ ± 9.62%	0.87 ± 1.08%

**Table 6:** Constant rates of reaction for the Zervos model based on DP, ε_cm,_ and σ_R_ for different insulation oils.

$(1/Property\;\left(DP,\;\varepsilon_{cm},\;\sigma_{R}\right)-(1/Property{\;}_{0}\;\left(DP,\;\varepsilon_{cm},\;\sigma_{R}\right))=(a/100)\times \left(2^{k\times t}-1\right)$						
	Mineral oil				Synthetic ester	
Temperature (°C)	k (DP)	a (DP)	R2	k (DP)	a (DP)	R2
150	−4.35 × 10^−3^ ± 1.22%	−1.07 ± 9.19%	0.99 ± 0.40%	−4.58 × 10^−3^ ± 9.39%	−3.02 × 10^−1^ ± 10.78%	0.98 ± 0.22%
130	−1.62 × 10^−3^ ± 0.99%	−8.89 × 10^−1^ ± 10.22%	0.96 ± 0.55%	−3.28 × 10^−3^ ± 3.35%	−1.69 × 10^−1^ ± 9.66%	0.96 ± 1.73%
110	−1.80 × 10^−4^ ± 9.43%	−2.75 × 10^−1^ ± 9.39%	0.97 ± 0.51%	−7.05 × 10^−4^ ± 10.63%	−1.41 × 10^−1^ ± 7.22%	0.94 ± 1.27%
Temperature (°C)	k (ε_cm_) _CD_	a (ε_cm_) _CD_	R2	k (ε_cm_) _CD_	a (ε_cm_) _CD_	R2
150	−2.89 × 10^−5^ ± 8.66%	−2.25 × 10^6^ ± 10.10%	0.89 ± 3.57%	−7.04 × 10^−3^ ± 7.84%	−7.39 × 10^3^ ± 8.27%	0.98 ± 1.08%
130	−1.72 × 10^−3^ ± 4.46%	−3.31 × 10^4^ ± 8.31%	0.97 ± 0.25%	−1.80 × 10^−3^ ± 8.59%	−2.24 × 10^3^ ± 3.68%	0.97 ± 0.60%
110	−2.37 × 10^−5^ ± 9.16%	−9.03 × 10^3^ ± 10.76%	0.99 ± 1.30%	−2.49 × 10^−4^ ± 10.19%	−1.01 × 10^3^ ± 1.70%	0.96 ± 1.90%
Temperature (°C)	k (ε_cm_) _MD_	a (ε_cm_) _MD_	R2	k (ε_cm_) _MD_	a (ε_cm_) _MD_	R2
150	−8.47 × 10^−4^ ± 4.98%	−1.41 × 10^5^ ± 10.32%	0.87 ± 0.41%	−2.32 × 10^−3^ ± 7.87%	−4.11 × 10^4^ ± 0.73%	0.87 ± 3.89%
130	−1.69 × 10^−3^ ± 3.25%	−6.88 × 10^4^ ± 4.74%	0.96 ± 0.49%	−2.44 × 10^−3^ ± 3.67%	−5.63 × 10^3^ ± 8.19%	0.99 ± 7.01%
110	−2.94 × 10^−4^ ± 7.77%	−4.38 × 10^3^ ± 10.22%	0.94 ± 4.05%	−7.86 × 10^−4^ ± 4.06%	−1.98 × 10^3^ ± 10.62%	0.91 ± 2.74%
Temperature (°C)	k (σ_R_) _CD_	a (σ_R_) _CD_	R2	k (σ_R_) _CD_	a (σ_R_) _CD_	R2
150	−1.89 × 10^−3^ ± 11.22%	−2.48 × 10^−2^ ± 3.52%	0.99 ± 1.05%	−9.21 × 10^−3^ ± 1.51%	−3.98 × 10^−3^ ± 6.80%	0.98 ± 3.70%
130	−1.74 × 10^−3^ ± 5.01%	−1.87 × 10^−2^ ± 11.96%	0.98 ± 1.355%	−2.91 × 10^−3^ ± 6.34%	−2.02 × 10^−3^ ± 4.63%	0.95 ± 1.73%
110	−1.44 × 10^−4^ ± 7.81%	−2.69 × 10^−3^ ± 5.14%	0.89 ± 7.73%	−4.52 × 10^−4^ ± 8.24%	−1.65 × 10^−3^ ± 10.26%	0.91 ± 2.07%
Temperature (°C)	k (σ_R_) _MD_	a (σ_R_) _MD_	R2	k (σ_R_) _MD_	a (σ_R_) _MD_	R2
150	−2.35 × 10^−3^ ± 5.20%	−1.63 × 10^−2^ ± 9.51%	0.90 ± 0.89%	−5.28 × 10^−5^ ± 8.69%	−1.87 × 10^−1^ ± 10.74%	0.83 ± 1.43%
130	−1.82 × 10^−3^ ± 3.56%	−1.28 × 10^−2^ ± 8.73%	0.97 ± 1.04%	−1.98 × 10^−3^ ± 9.37%	−1.12 × 10^−3^ ± 5.03%	0.99 ± 1.50%
110	−1.10 × 10^−4^ ± 10.41%	−1.73 × 10^−3^ ± 3.32%	0.98 ± 1.57%	−1.29 × 10^−4^ ± 9.77%	−1.20 × 10^−3^ ± 2.24%	0.87 ± 1.12%

**Table 7:** Constant rates of reaction for the Calvini model based on DP, ε_cm,_ and σ_R_ for different insulation oils.

$(Property{\;}_{0}\;\left(DP,\;\varepsilon_{cm},\;\sigma_{R}\right)/Property\;\left(DP,\;\varepsilon_{cm},\;\sigma_{R}\right))-1=(Property{\;}_{0}/LOProperty)\times \left(1-exp\left(-k\times t\right)\right)$				
	Mineral oil		Synthetic ester	
Temperature (°C)	k (DP)	R2	k (DP)	R2
150	1.23 × 10^−2^ ± 3.64%	0.25 ± 3.82%	9.69 × 10^−4^ ± 0.54%	0.88 ± 4.77%
130	2.97 × 10^−3^ ± 1.65%	0.75 ± 1.71%	2.27 × 10^−4^ ± 1.43%	0.62 ± 2.47%
110	3.98 × 10^−5^ ± 3.85%	0.92 ± 0.67%	2.69 × 10^−5^ ± 3.03%	0.21 ± 4.27%
Temperature (°C)	k (ε_cm_) _CD_	R2	k (ε_cm_) _CD_	R2
150	5.10 × 10^−1^ ± 4.75%	<0.01 ± 8.10%	9.85 × 10^−3^ ± 2.15%	0.78 ± 3.17%
130	7.68 × 10^−3^ ± 3.25%	<0.01 ± 4.20%	2.61 × 10^−4^ ± 1.62%	0.85 ± 3.24%
110	2.83 × 10^−5^ ± 5.84%	0.99 ± 1.05%	1.33 × 10^−5^ ± 1.44%	0.80 ± 8.22%
Temperature (°C)	k (ε_cm_) _MD_	R2	k (ε_cm_) _MD_	R2
150	7.87 × 10^−1^ ± 3.98%	<0.01 ± 1.12%	7.41 × 10^−1^ ± 7.70%	0.29 ± 4.98%
130	8.63 × 10^−3^ ± 4.73%	<0.01 ± 9.31%	3.53 × 10^−4^ ± 6.39%	0.80 ± 1.70%
110	3.18 × 10^−5^ ± 5.06%	0.75 ± 9.71%	1.57 × 10^−5^ ± 8.33%	0.11 ± 7.11%
Temperature (°C)	k (σ_R_) _CD_	R2	k (σ_R_) _CD_	R2
150	9.61 × 10^−1^ ± 9.37%	0.05 ± 6.41%	4.75 × 10^−3^ ± 6.15%	0.48 ± 9.56%
130	3.38 × 10^−3^ ± 5.19%	0.61 ± 2.07%	1.27 × 10^−4^ ± 5.34%	0.61 ± 7.18%
110	1.57 × 10^−5^ ± 4.79%	0.85 ± 10.20%	1.41 × 10^−5^ ± 6.94%	0.51 ± 8.80%
Temperature (°C)	k (σ_R_) _MD_	R2	k (σ_R_) _MD_	R2
150	6.68 × 10^−3^ ± 9.71%	0.56 ± 9.64%	7.15 × 10^−4^ ± 4.56%	0.56 ± 12.23%
130	5.90 × 10^−3^ ± 1.43%	0.36 ± 5.18%	1.92 × 10^−4^ ± 9.69%	0.82 ± 3.84%
110	2.63 × 10^−5^ ± 11.59%	0.96 ± 1.05%	1.88 × 10^−5^ ± 5.49%	0.83 ± 1.06%

**Table 8:** Coefficients of aging for the Weidmann model based on DP, ε_cm,_ and σ_R_ for different insulation oils.

$Property\;\left(DP,\;\varepsilon_{cm},\;\sigma_{R}\right)=Property{\;}_{0}\;\left(DP,\;\varepsilon_{cm},\;\sigma_{R}\right)\times exp\left(-C_{property}\;\left(T\right)\times t\right)$				
	Mineral oil		Synthetic ester	
Temperature (°C)	C_DP_	R2	C_DP_	R2
150	2.89 × 10^−3^ ± 10.27%	0.31 ± 1.90%	1.51 × 10^−3^ ± 10.04%	0.45 ± 4.69%
130	1.12 × 10^−3^ ± 9.60%	0.41 ± 6.30%	4.81 × 10^−4^ ± 9.20%	0.21 ± 11.35%
110	7.28 × 10^−5^ ± 1.79%	0.59 ± 1.24%	5.91 × 10^−5^ ± 2.48%	<0.01 ± 9.26%
Temperature (°C)	C_εcm CD_	R2	C_εcm CD_	R2
150	4.38 × 10^−3^ ± 9.57%	0.51 ± 2.97%	2.56 × 10^−3^ ± 9.08%	0.15 ± 10.81%
130	1.86 × 10^−3^ ± 9.52%	0.39 ± 3.32%	5.17 × 10^−4^ ± 9.17%	0.61 ± 6.49%
110	6.03 × 10^−5^ ± 6.53%	0.96 ± 1.08%	3.74 × 10^−5^ ± 2.11%	0.71 ± 8.49%
Temperature (°C)	C_εcm MD_	R2	C_εcm MD_	R2
150	3.93 × 10^−3^ ± 9.27%	0.31 ± 1.30%	3.22 × 10^−3^ ± 9.83%	0.30 ± 1.70%
130	1.83 × 10^−3^ ± 9.54%	0.32 ± 4.41%	5.98 × 10^−4^ ± 7.05%	0.38 ± 10.08%
110	6.43 × 10^−5^ ± 6.75%	0.53 ± 10.83%	4.22 × 10^−5^ ± 4.14%	<0.01 ± 4.20%
Temperature (°C)	C_σR CD_	R2	C_σR CD_	R2
150	2.66 × 10^−3^ ± 8.03%	0.56 ± 6.23%	1.31 × 10^−3^ ± 6.07%	0.16 ± 9.48%
130	1.19 × 10^−3^ ± 4.98%	0.47 ± 2.70%	3.36 × 10^−4^ ± 6.29%	0.45 ± 6.57%
110	4.21 × 10^−5^ ± 8.96%	0.68 ± 6.22%	3.91 × 10^−5^ ± 7.35%	0.21 ± 4.46%
Temperature (°C)	C_σR MD_	R2	C_σR MD_	R2
150	3.60 × 10^−3^ ± 9.07%	0.27 ± 8.66%	2.49 × 10^−3^ ± 8.63%	0.65 ± 2.26%
130	1.56 × 10^−3^ ± 7.68%	0.39 ± 1.28%	4.36 × 10^−4^ ± 9.25%	0.58 ± 9.59%
110	5.81 × 10^−5^ ± 2.60%	0.83 ± 1.66%	4.73 × 10^−5^ ± 10.49%	0.59 ± 6.65%

**Table 9:** Constants for the IEEE model based on lifetime needed to reach end-of-life criteria (DP = 350, ε_cm_ = 0.5 ∗ ε_cm0_, σ_R_ = 0.5 ∗ σ_R0_) for different insulation oils.

$\ln L\left(T\right)=A+\left(B/T\right)$					
Mineral oil			Synthetic ester		
A (DP)	B (DP)	R2	A (DP)	B (DP)	R2
9.16 × 10^−17^ ± 1.15%	17,358 ± 2.05%	0.92 ± 1.08%	9.17 × 10^−18^ ± 2.85%	18,715 ± 2.75%	0.99 ± 1.35%
A (ε_cm_) _CD_	B (ε_cm_) _CD_	R2	A (ε_cm_) _CD_	B (ε_cm_) _CD_	R2
2.79 × 10^−18^ ± 3.25%	18,648 ± 1.65%	0.89 ± 0.95%	1.01 × 10^−19^ ± 1.15%	21,883 ± 0.75%	0.96 ± 0.85%
A (ε_cm_) _MD_	B (ε_cm_) _MD_	R2	A (ε_cm_) _MD_	B (ε_cm_) _MD_	R2
6.55 × 10^−16^ ± 2.65%	16,344 ± 4.20%	0.82 ± 1.11%	1.57 × 10^−21^ ± 3.15%	21,916 ± 0.97%	0.94 ± 2.07%
A (σ_R_) _CD_	B (σ_R_) _CD_	R2	A (σ_R_) _CD_	B (σ_R_) _CD_	R2
2.55 × 10^−20^ ± 1.15%	20,637 ± 2.15%	0.87 ± 2.07%	2.60 × 10^−18^ ± 2.95%	18,982 ± 1.65%	0.96 ± 4.24%
A (σ_R_) _MD_	B (σ_R_) _MD_	R2	A (σ_R_) _MD_	B (σ_R_) _MD_	R2
3.09 × 10^−20^ ± 3.65%	20,587 ± 3.07%	0.90 ± 1.19%	3.64 × 10^−19^ ± 4.08%	19,999 ± 3.95%	0.99 ± 2.67%

## Data Availability

Data is contained within the article.
